# Visualizing the replicating HSV-1 virus using STED super-resolution microscopy

**DOI:** 10.1186/s12985-016-0521-7

**Published:** 2016-04-09

**Authors:** Zhuoran Li, Ce Fang, Yuanyuan Su, Hongmei Liu, Fengchao Lang, Xin Li, Guijun Chen, Danfeng Lu, Jumin Zhou

**Affiliations:** Key Laboratory of Animal Models and Human Disease Mechanisms of Chinese Academy of Sciences & Yunnan Province, Kunming Institute of Zoology, Chinese Academy of Sciences, NO. 32 Jiaochang Donglu, Kunming, Yunnan 650223 People’s Republic of China; University of Chinese Academy of Sciences, Beijing, 100049 People’s Republic of China; Leica Microsystems Trading Limited, Shanghai, 201206 People’s Republic of China

**Keywords:** HSV-1 replication, STED, IF, FISH, RNA Pol II, ICP8

## Abstract

**Background:**

Replication of viral genome is the central event during the lytic infectious cycle of herpes simplex virus 1 (HSV-1). However, the details of HSV-1 replication process are still elusive due to the limitations of current molecular and conventional fluorescent microscopy methods. Stimulated emission depletion (STED) microscopy is one of the recently available super-resolution techniques allowing observation at sub-diffraction resolution.

**Methods:**

To gain new insight into HSV-1 replication, we used a combination of stimulated emission depletion microscopy, fluorescence in situ hybridization (FISH) and immunofluorescence (IF) to observe the HSV-1 replication process.

**Results:**

Using two colored probes labeling the same region of HSV-1 genome, the two probes highly correlated in both pre-replication and replicating genomes. In comparison, when probes from different regions were used, the average distance between the two probes increased after the virus enters replication, suggesting that the HSV-1 genome undergoes dynamic structure changes from a compact to a relaxed formation and occupies larger space as it enters replication. Using FISH and IF, viral single strand binding protein ICP8 was seen closely positioned with HSV-1 genome. In contrast, ICP8 and host RNA polymerase II were less related. This result suggests that ICP8 marked regions of DNA replication are spatially separated from regions of active transcription, represented by the elongating form of RNA polymerase II within the viral replication compartments. Comparing HSV-1 genomes at early stage of replication with that in later stage, we also noted overall increases among different values. These results suggest stimulated emission depletion microscopy is capable of investigating events during HSV-1 replication.

**Conclusion:**

1) Replicating HSV-1 genome could be observed by super-resolution microscopy; 2) Viral genome expands spatially during replication; 3) Viral replication and transcription are partitioned into different sub-structures within the replication compartments.

## Background

**Fig. 1:**
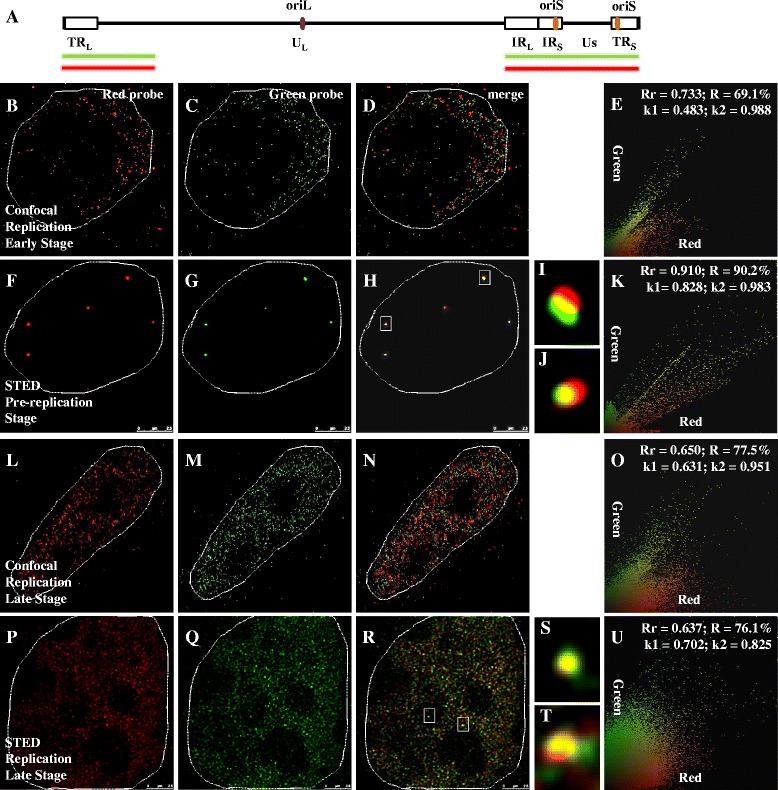
Resolution of STED microscopy is higher than confocal microscopy. All cells were infected with HSV-1 17+ strain for 6 h, then prepared for FISH. In first line, signals are captured from red channel, which were hybridized with Biotin labeled probe; Second line, signals are captured from green channel, which were hybridized with DIG labeled probe; Third line, images are merged to examine colocalization situation of two color signals; Fourth line, partial enlarged detail of figures in the third line are shown; Fifth line, images from the third line were analyzed, which were done with Image-Pro Plus 6.0 software (USA). **a**: A brief description of HSV-1 genome structure. Relative to HSV-1 genome, probe locates at the two terminus, which contains TR_L_, IR_L_, IR_S_, TR_S_, U_S_ and partial U_L_ region. The same probe is labeled with either DIG or Biotin to generate two different colors. **b**-**e**, **f**-**k**: Cells were infected at a MOI of 0.1 PFU/cell. At early stage of HSV-1 replication, images are captured with confocal microscopy and STED microscopy, respectively, and then analyzed. **l**-**o**, **p**-**u**: Cells were infected at a MOI of 5 PFU/cell. At late stage of HSV-1 replication, images are captured with confocal microscopy and STED microscopy, respectively, and then analyzed. Host cell nucleus are indicated with white dotted lines. **i**, **j**, **s**, **t**: Higher zooms of regions inside the white rectangles are shown. Scale bars, 2.5 μm. Rr: correlation coefficient; R: overlapping coefficient; *k*: antigen contribution

HSV-1, a virus of the *Herpesviridae* family [1], possesses a linear double-stranded 152-kbp genome with three origins of DNA replication and approximately 75 open-reading frames [2]. HSV-1 is a common but important human pathogen, infecting more than 80 % of the population, resulting in life-long recurrent disease in a third of infected individuals [3, 4]. The HSV-1 genome consists of unique and repeated sequences (Fig. 1a), with two covalently joined segments, L and S, each comprises a unique region (U_L_ and U_S_) flanked by a set of inverted repeats (TR_L_ and IR_L_, TR_S_ and IR_S_, respectively) [1]. Following viral infection and entry of epithelial cell *in vivo*, the HSV-1 genome is released into the host nucleus and initiates lytic infection (productive infection), after which virus can infect innervating axons of sensory neurons and establish latent infections in the peripheral nervous system [5, 6]. The former is characterized by active expression of almost all viral genes in a highly ordered temporal cascade, while the latter is characterized by restricted viral gene expression, the absence of viral DNA synthesis and infectious virus.

The HSV-1 genome contains three origins of DNA replication: one copy of oriL (purple oval) located at the center of the U_L_ region and two copies of oriS (orange oval) located in repeated sequences flanking the U_S_ region (Fig. 1a) [7]. Upon entering the cell nucleus, the linear viral genome circularizes and DNA replication initiates at these origins. Two competing hypotheses exist to account for the mode of replication. In the linear replication model, circular genomes do not form wild-type virus, which is supported by a study using the Gardella gel method [8]. The circular model proposes that the replication initially proceeds by a theta mechanism and subsequently switches to a sigma or rolling-circle form to yield long head-to-tail concatemers. This model is supported by restriction enzyme digestion experiments [1, 2, 9, 10]. Electron microscopy detected extensive regions of single-stranded DNA, DNA replication forks, loops, and branched DNA structures [11–13].

Replication of viral genome is a central, well orchestrated event of HSV-1 lytic infection, which leads to the development of viral replication compartments or centers---structures consisted of replicating viral genomes and many viral proteins (UL5, UL8, UL9, UL29, UL30, UL42 and UL52) and cellular proteins [5, 14–18]. In particular, HSV-1 single-strand DNA-binding protein or infected cell protein 8 (ICP8) [19] encoded by the *UL29* gene [20–22], interacts with host cell nuclear matrix and viral single strand DNA in its maturational process, and is required for viral replication [23]. Approximately half of the HSV-1 genomic DNA becomes soluble at 2 h post-infection and most of HSV-1 DNA is in unstable nucleosome-like complexes throughout the lytic replication stage, suggesting a dynamic nature of viral genome during replication [5, 18, 24, 25].

Though extensive studies were conducted on HSV-1 replication [1, 2, 5, 7–13, 17, 18, 24, 26–28], there is still a lack of direct and effective method to observe the structural changes of viral genome during replication.

STED microscopy is one of the recent techniques that accomplish super-resolution microscopy with optimal for lateral and axial resolutions at 16–40 nm and < 80 nm in the focal plane, respectively [29–31]. It is developed by Stefan W. Hell and Jan Wichmann in 1994 [32], and firstly applied in experiments in 1999, that is implemented by Thomas Klar and Stefan W. Hell. Hell was awarded the Nobel Prize in Chemistry in 2014 for his contribution to the STED microscopy. STED microscopy creates super-resolution images by the selective deactivation of fluorophores, minimizing the area of illumination at the focal point, and thus enhancing the achievable resolution for a given system [33].

Here we used FISH or IF-FISH technique with STED microscopy to visualize HSV-1 genome and interacting proteins during viral replication. We found that the viral genome appeared to become relaxed, as it occupied larger space after it initiated DNA synthesis in the host nucleus, with the average distance between the two probes designed to hybridize to neighboring regions of the viral genome increased by 2.7-fold. Using FISH and IF, we showed that the ICP8 protein interacted with the viral genome with high colocalization coefficient (m2), and it appeared to be organized in different sub-structures from that of RNA polymerase II (RNA Pol II) based on staining patterns and its distance from RNA Pol II, suggesting that DNA replication and transcription are likely carried out in distinct regions within the replication compartments.

## Results

### STED microscopy can reliably detect the viral genome

To examine how STED and confocal microscopy differ, we labeled DNA probes designed towards the terminal regions of the viral genome (Fig. 1a) with either DIG (green) or Biotin (red) to generate two different colored probes to the same region of the viral genome to determine. The human primary fibroblast cells (BJ cells) were infected with the 17+ strain of HSV-1 at multiplicity of infection (MOI) of 0.1 or 5 PFU/cell for 6 h. Due to the heterogeneity of cells and variation in the number of incoming viruses in each cell, viral replication time varies from one cell to another, and as a result, progressing from small but distinct early replication compartments to large fused late replication compartments occupying most of the host nucleus took about 6 h post-infection. At a lower MOI (0.1 PFU/cell), we observed more smaller replication compartments, while at a high MOI of 5 PFU/cell infection, larger fused compartments were typically observed [14].

Confocal microscopy was developed to offer greater resolution than regular fluorescent microscopes by rejection of out-of-focus noise [34, 35]. Fig. 1b-d were captured with confocal microscopy to show BJ cells at early stage of replication. Fig. 1b, c were from red and green channels, respectively. Figure 1d is an overlay of Fig. 1b and c, while Fig. 1e stands for the analysis results of Fig. 1d. Correlation coefficient (Rr), also known as Pearson’s correlation coefficient, ranges from −1.0 to 1.0. 0 indicates no correlation between two signals and −1.0 represents complete negative correlation. Overlapping coefficient (R) represents the colocalization frequency of two selected signals [36]. The Rr and R of Fig. 1d are 0.733 and 69.1 %, respectively (Fig. 1e), suggesting a moderate correlation between the two probes.

STED microscopy results were shown in Fig. 1f-h. Figure 1f, g were from red and green channels, respectively, Fig. 1h is overlay of Fig. 1f and Fig. 1g. While Fig. 1i, j are details with enlargement of partial Fig. 1h, which are indicated by white rectangles. Figure 1k stands for the analysis results of Fig. 1h. Unlike confocal microscopy, there is a much better overlap between red and green signals from STED (Fig. 1h). The center sections of the two color signals overlapped tightly (Fig. 1i, j). The Rr of the two signals is 0.910, and R is 90.2 % (Fig. 1k). Values are much higher than that from confocal results. The visual colocalization and the high values of Rr and R from STED analysis demonstrate that STED is able to detect viral genomes.

To determine how these probes behave at the late stage of viral replication compartments development, when individual replication compartments merges into large ones occupying most of the host nucleus, we infected BJ cells at a high MOI of 5 PFU/cell for 6 h and examined the signals by confocal (Fig. 1l-n) and STED microscopy (Fig. 1p-r). Figure 1l (red signal), 1 M (green signal) are merged in Fig. 1n and related parameters are shown in Fig. 1o. Though the Rr and R of confocal image Fig. 1n are 0.650 and 77.5 % (Fig. 1o), respectively, there is still no macroscopic overlapping between two signals under the confocal microscopy, indicating that confocal microscopy again failed to convincingly colocalize the two signals.

In contrast, Fig. 1p (red probe) and 1Q (green probe) exhibite stronger correlations when merged in Fig. 1r and analyzed in Fig. 1u. Figure 1s, t are details with enlargement of partial Fig. 1r (white rectangles) to show overlapping red and green signals. In Fig. 1s, two color signals overlapped completely, and in Fig. 1t, just part of the signals overlapped. Under the STED microscopy, about 76.1 % of the two color signals overlapped (Fig. 1r). The Rr of Fig. 1r is 0.637 (Fig. 1u). Comparing Fig. 1h and Fig. 1r, both Rr and R decrease with the development of replication compartments.

As each DNA strand of the viral genome stochastically hybridize to red or green probes, the chances of a perfect overlap between red and green signals is approximately 25 % when there is abundant amount of probes present, such as at early stage of replication compartments development. In cells where viral replication compartments are well developed, there are a larger number of viral genomes, and a limited amount of probes present, which would result in an increased possibility of only one colored probe hybridizing to a single viral genome, thus the observed reduction of overlapping signals, and hence the decrease in Rr and R from STED imaging. The lack of changes in the Rr and *R* values from confocal imaging suggests that the confocal microscopy is intrinsically unreliable to describe the details needed for HSV-1 genomes.

### Replication renders compact HSV-1 genomes into relaxed structures

**Fig. 2:**
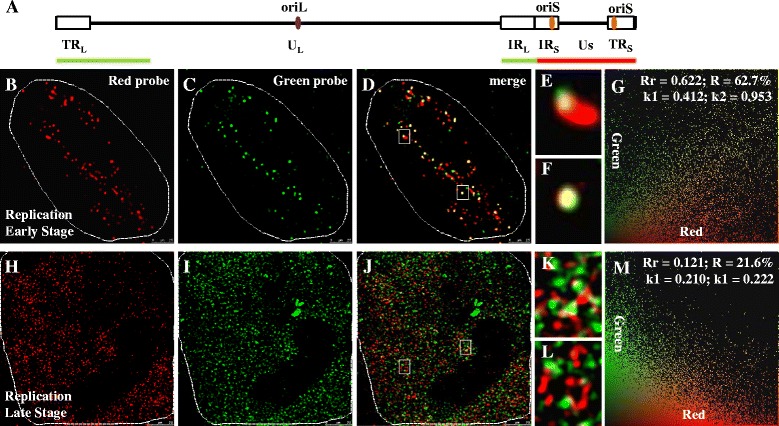
Replication renders compact HSV-1 genomes into relaxed structures. All cells were infected with HSV-1 17+ strain for 6 h, then prepared for FISH. In first line, signals are captured from red channel, which were hybridized with Biotin labeled probe; Second line, signals are captured from green channel, which were hybridized with DIG labeled probe; Third line, images are merged to examine the colocalization situation of two color signals; Fourth line, partial enlarged detail of figures in the third line are shown; Fifth line, images from the third line were analyzed, which were done with Image-Pro Plus 6.0 sofrware (USA). **a**: A brief description of HSV-1 genome structure. Relative to HSV-1 genome, red probe labeled with Biotin locates at the right terminal, which contains IR_S_, TR_S_ and U_S_ region (according to NC_001806.2 127235–131131, 132647–133909, 134056–134931, 135225–136670, 136747–137463, 138423–139607, 139789–140961, 141247–142899 and 147066–150962). Green probe labeled with DIG locates at the right terminal, which contains TR_L_, IR_L_ and partial U_L_ region (according to NC_001806.2, 513–1259, 2262–2318, 3084–3750, 3887–5490, 9338–10012, 10991–11665, 12484–15132, 151131–17161, 18225–20477, 20705–23260, 120884–122487, 122624–123290, 124056–124112 and 125115–125861). **b**-**g**: Cells were infected at a MOI of 0.1 PFU/cell. At early stage of HSV-1 replication, images are captured with STED microscopy and then analyzed. **h**-**m**: Cells were infected at a MOI of 5 PFU/cell. At late stage of HSV-1 replication, images are captured with STED microscopy and then analyzed. Host cell nucleus are indicated with white dotted lines. **e**, **f**, **k**, **l**: Higher zooms of regions inside the white rectangles are shown. Scale bars, 2.5 μm. Rr: correlation coefficient; R: overlapping coefficient; *k*: antigen contribution

When HSV-1 DNA enters the host nucleus, it assumes a condensed structure, with a diameter of 35–40 nm and a length of 130–160 nm [37]. The interaction between HSV-1 genome and host core histones occurs as early as 1 h post-infection, and the viral genome forms a nucleosome-like structure. Unlike the viral genome at the pre-replication stage, most of the replicating HSV-1 genome is in a nucleosome-free state [24], and likely assumes a less condensed structure. The nucleosome-like HSV-1 genome is unstable and the accessibility to micrococcal nuclease (MNase) changes throughout the replication process. HSV-1 DNA is quantitatively recovered in complexes fractionating as mono- to polynucleosomes from nuclei harvested at 2, 5, 7, or 9 h post-infection. At 1 h post-infection, the whole HSV-1 genome is in nucleosomal stage and, at 2, 5, 7, or 9 h post-infection the viral genome lose nucleosome in different levels, suggested the stability of HSV-1 DNA nucleosomal complexes changes throughout the lytic infection cycle [5, 18, 24, 25]. To directly observe the dynamic structural changes in the HSV-1 replication process, probes were designed to recognize the termini of the viral genome (Fig. 2a). The two probes were labeled with either DIG or Biotin to give them two different colors.

**Fig. 3:**
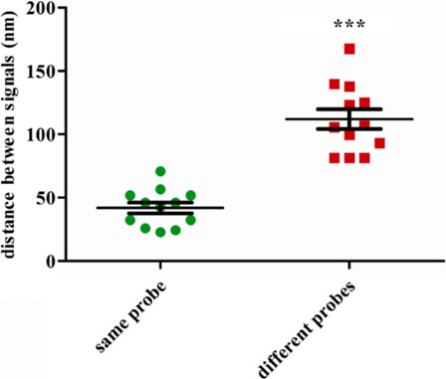
Average distances of the same probe and different probes. Distances of the same probe and different probes were calculated under STED microscopy. The average distance of the same probe is 41.9 nm and that of different probes is 111.9 nm, which is 2.7-fold higher than the same probe, *p* value < 0.001 (***). The data were evaluated with the Students’ t-test

BJ cells were infected at a low MOI of 0.1 PFU/cell and were processed for STED microscopy at the early stage of viral replication. Figure 2b (red) and 2C (green) are merged in Fig. 2d to show how the two colored signals relate. Pearson analysis of Fig. 2g shows that most of signals overlapped under STED microscopy (Fig. 2d), The Rr and R are 0.622 and 62.7 % (Fig. 2g), respectively. Parts of Fig. 2d (white rectangles) are enlarged to reveal two typical examples (Fig. 2e, f), where the red and green signals are directly connected or overlap. As Fig. 2e shows, the green signal is connected with the red oblong signal, but in Fig. 2f, the two colors sit right on top of each other. This is likely a result of differences in viral genome orientation. Compared with the correlation between two colored probes directed to the same region of the viral genome, the two probes directed toward different regions of the viral genome shows significantly lower correlation than the probes from the same region (compare Fig. 1h, k and 2d, g). The average distance between the two color signals from the same probe is 41.9 nm, but that of different probes is 111.9 nm, 2.7-fold higher (Fig. 3). These results suggest that STED microscopy is able to distinguish different regions of the viral genome at early stage of replication.

We next measured the distance between the different regions of HSV-1 genome in fully developed replication compartments. Signals in Fig. 2h (red) and Fig. 2i (green) are merged in Fig. 2j, and Pearson analysis is shown in Fig. 2m. Unlike the early stage of replication, viral genomes in advanced replication compartments do not show overlap and display very low correlation between the red and green signals (Fig. 2j). The Rr and R of Fig. 2j are 0.121 and 21.6 % (Fig. 2m), respectively, indicating very low correlations. Parts of Fig. 2j, which are indicated by white rectangles, are enlarged to reveal two typical examples (Fig. 2k, l), where we could see that the red and green probes detected elongated, fiber like structures.

In Fig. 3, the average distance between the two color signals from the same probe is 41.9 nm with a range from 22.6 nm to 70.8 nm, where as that of different probes is 111.9 nm with a range from 81.4 nm to 167.6 nm. At the pre-replication stage or early stage of replication, both the distances between the two color probes directed towards the same region, and the two probes, directed to different regions are relatively small. But, as viral replication progresses, these distances become greater. These results (Figs. 1, 2 and 3) suggest that pre-replication and early replication HSV-1 genomes exist as compact structures, while viral genomes in later replication compartments assume relaxed structures occupying significantly large space.

### The ICP8 signals is highly related to the replicating HSV-1 genome

ICP8 interacts with the replicating parts of the viral genome and is used as a marker of HSV-1 replication. It also possesses multiple functions to facilitate viral replication and regulate viral genes expression [20, 22, 38, 39]. We therefore examined the distribution of ICP8 during replication to reveal the dynamic changes in the HSV-1 genomes.

**Fig. 4:**
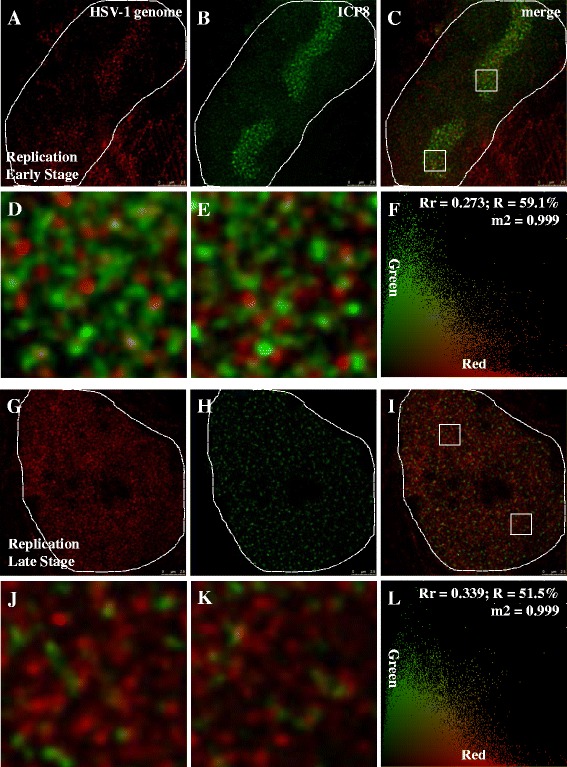
ICP8 signals is highly related to the replicating HSV-1 genome. All cells were infected with HSV-1 17+ strain and at a MOI of 5 PFU/cell for 6 h, then prepared for IF-FISH. **a**-**c**: At early stage of HSV-1 replication, images are captured with STED microscopy. **d**, **e**: Higher zooms of regions inside C are shown, which are indicated by white squares. **f**: Analysis results of C is shown. **g**-**i**: At late stage of HSV-1 replication, images are captured with STED microscopy. **j**, **k**: Higher zooms of regions inside I are shown, which are indicated by white squares. **l**: Analysis results of I is shown. Host cell nucleus are indicated with white dotted lines. Scale bars, 2.5 μm. Rr: correlation coefficient; R: overlapping coefficient; m2: colocalization coefficient

Again, BJ cells were infected at a high MOI of 5 PFU/cell for 6 h and HSV-1 genomes were detected by FISH using labeled BAC clone probe covering the entire HSV-1 genome. As shown in the analysis in Fig. 4, ICP8 IF signals are tightly colocalized or associated with HSV-1 genome at both early (Fig. 4c) and late stages of replication (Fig. 4i). Colocalization coefficient (m2) describes contribution of positive staining pixels from each selected channels [36]. The value of m2 in Fig. 4c and Fig. 4i are 0.999 for both (Fig. 4f, l), indicating that 99.9 % green (ICP8) colocalize with red pixels (HSV-1 genome) in these figures. Figure 4d and e show local enlargements of the two white squares (Fig. 4c) to reveal visually the red and green signals are closely associated. As viral replication compartments became larger, ICP8 positive areas also grew with the compartments to eventually occupy the whole host nucleus (Fig. 4h). While the Rr and R of early stage of replication are 0.273 and 59.1 %, respectively, those of late stage of replication are 0.339 and 51.5 %, respectively. From a comparison between Fig. 4d and j, we could note an increase of viral genome signals and a reduction of ICP8 signals. This is because, at the early stage of replication, the infected nucleus has a large reserve of ICP8 proteins to prepare for replication, and viral genomes are in a smaller number. While, at the late stage of replication, the situation is reversed, with a huge number of viral genomes and a relative smaller amount of ICP8 proteins in the host cell nucleus. Consequently, at the early stage, the *Rr* value is lower than that at late stage of replication. With the development of replication compartments, the structure of the viral genome becomes more and more relaxed, and the average distance between ICP8 protein and the HSV-1 genome changes from 132.4 nm to 183.6 nm, *p* value < 0.001 (Fig. 7). Thus, R decreases with the replication progress from early to late stage.

### ICP8 occupies sub-structures within the viral replication compartments distinct from host RNA Pol II

Molecular and immunofluorescent studies suggest that HSV-1 replication and viral gene transcription are both occurring within the viral replication compartments [40]. However, transcription and DNA replication are two incompatible processes, i.e. the same region of the genome is difficult to replicate and transcribe at the same time [41]. Viral proteins for HSV-1 replication and viral genes are all transcribed by host RNA Pol II [42, 43]. RNA Pol II is regulated by phosphorylation of its carboxyl-terminal domain (CTD), with modification occurring primarily on serine 2 and 5 of the CTD. The serine 2 phosphorylated form of RNA Pol II (RNA Pol II Ser2P) is mostly associated with elongating form and active transcription, while the serine 5 phosphorylated form (RNA Pol II Ser5P) is more related to paused polymerase [44].

**Fig. 5:**
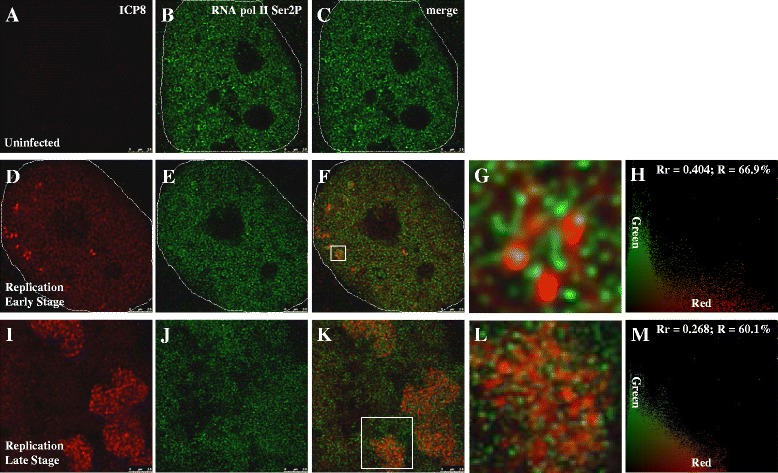
Double immunostaining of ICP8 and RNA Pol II Ser2P. Experimental group cells were infected with HSV-1 17+ strain for 6 h, then fixed for IF. In first line, signals are captured from red channel, which were stained with anti-ICP8 monoclonal antibody; Second line, signals are captured from green channel, which were stained with anti-RNA Pol II Ser2P polyclonal antibody; Third line, images are merged to examine colocalization situation of two color signals; Fourth line, partial enlarged detail of figures in the third line are shown; Fifth line, images from the third line were analyzed, which were done with Image-Pro Plus 6.0 software (USA). **a**-**c**: Cells were not infected, images are captured with STED microscopy. **d**-**h**: Cells were infected at a MOI of 0.1 PFU/cell, images are captured with STED microscopy and then analyzed. **i**-**m**: Cells were infected at a MOI of 5 PFU/cell, images are captured with STED microscopy and then analyzed. Host cell nucleus are indicated with white dotted lines. **g**, **l**: Higher zooms of regions inside the white squares are shown. Scale bars, 2.5 μm. Rr: correlation coefficient; R: overlapping coefficient

To determine how the ICP8 staining signals is related to RNA Pol II, we firstly performed double immunostaining using anti-ICP8 monoclonal antibody (Fig. 5a, d, i) and anti-RNA Pol II Ser2P polyclonal antibody (Fig. 5b, e, j). The images are merged to examine the colocalization of two color signals. As shown in Fig. 5f, there is a slight but visible increase of the RNA Pol II Ser2P colocalized with ICP8 marked early replication compartments. Local enlargement (Fig. 5g) shows that these two signals are related but do not overlap. The Rr and R of Fig. 5f are 0.404 and 66.9 % (Fig. 5h), respectively.

To observe well developed replication compartments, cells were infected at a high MOI of 5 PFU/cell for 6 h prior to fixing for IF analysis. In these cells (Fig. 5i), RNA Pol II Ser2P evenly distributed, with a slight enrichment in areas overlapping with the ICP8 labeled replication compartments (Fig. 5j). Again ICP8 and RNA Pol II Ser2P do not show obvious overlap (Fig. 5k). The *Rr* value of Fig. 5k is 0.268, and the *R* value is 60.1 % (Fig. 5m). The average distances between ICP8 and RNA Pol II Ser2P at early and late stages of replication are 262.2 nm and 283.0 nm, respectively, and the difference between these two is not significant, *p* value > 0.05 (Fig. 7). These results suggest that ICP8 and RNA Pol II Ser2P do not show significant association.

**Fig. 6:**
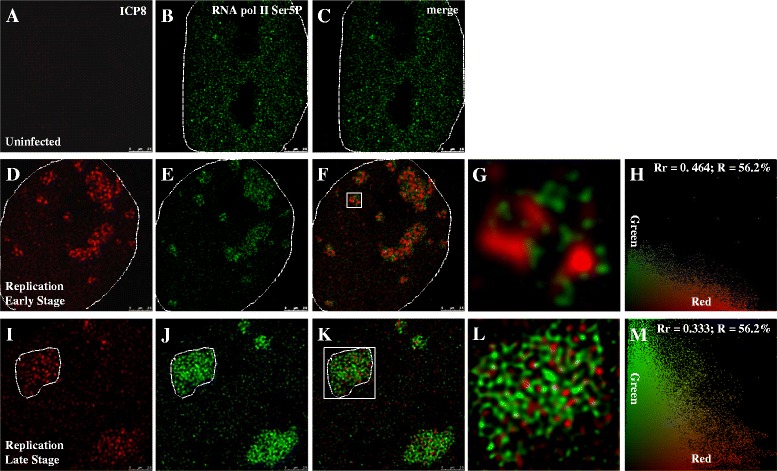
Double immunostaining of ICP8 and RNA Pol II Ser5P. Experimental group cells were infected with HSV-1 17+ strain for 6 h, then fixed for IF. In first line, signals are captured from red channel, which were stained with anti-ICP8 monoclonal antibody; Second line, signals are captured from green channel, which were stained with anti-RNA Pol II Ser5P polyclonal antibody; Third line, images are merged to examine colocalization situation of two color signals; Fourth line, partial enlarged detail of figures in the third line are shown; Fifth line, images from the third line were analyzed, which were done with Image-Pro Plus 6.0 software (USA). **a**-**c**: Cells were not infected, images are captured with STED microscopy. **d**-**h**: Cells were infected at a MOI of 0.1 PFU/cell, images are captured with STED microscopy and then analyzed. **i**-**m**: Cells were infected at a MOI of 5 PFU/cell, images are captured with STED microscopy and then analyzed. Host cell nucleus are indicated with white dotted lines. **g**, **l**: Higher zooms of regions inside the white squares are shown. Scale bars, 2.5 μm. Rr: correlation coefficient; R: overlapping coefficient

ICP8 and RNA Pol II Ser5P double staining were conducted, but unlike RNA Pol II Ser2P, RNA Pol II Ser5P showed stronger colocalization in the viral replication compartments at 6 h post-infection at a low MOI of 0.1 PFU/cell and at the early stage of replication (Fig. 6f). The Rr and R of Fig. 6f are 0.464 and 56.2 % (Fig. 6h), respectively. When cells were infected at a high MOI of 5 PFU/cell and at the late stage of replication, RNA Pol II Ser5P still colocalizes with ICP8 (Fig. 6k). The Rr and R of Fig. 6k are 0.333 and 56.2 % (Fig. 6m), respectively.

**Fig. 7:**
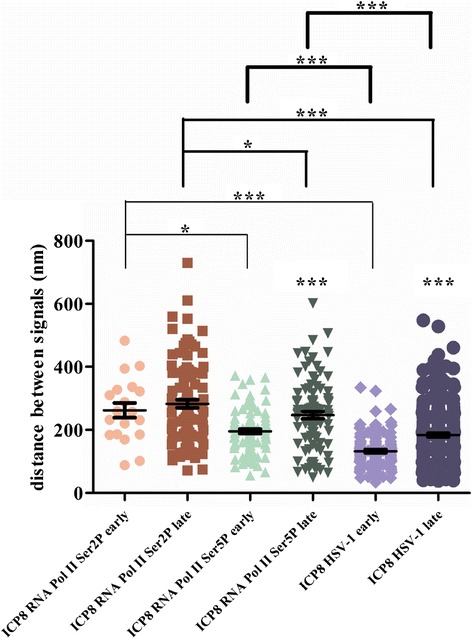
Average distances of ICP8 and RNA Pol II Ser2P, ICP8 and RNA Pol II Ser5P, HSV-1 genome and ICP8. At early stage of replication, average distances of ICP8 and RNA Pol II Ser2P, ICP8 and RNA Pol II Ser5P, ICP8 and HSV-1 genome are 262.2 nm, 195.7 nm and 132.4 nm, respectively. Similarly, average distances of late stage of replication are 283.0 nm, 247.0 nm and 183.6 nm. Differences between early and late stages of replication (ICP8 and RNA Pol II Ser5P, ICP8 and HSV-1 genome) are significant (*p* value_ICP8 RNA Pol II Ser5P (early and late stage)_ < 0.001 (***), *p* value_ICP8 HSV-1 genome(early and late stage)_ < 0.001 (***)). Differences among ICP8 and RNA Pol II Ser2P, ICP8 and RNA Pol II Ser5P, ICP8 and HSV-1 genome are all significant (*p* value_ICP8 RNA Pol II Ser2P and ICP8 RNA Pol II Ser5P (early and late stage)_ < 0.05 (*), *p* value_ICP8 RNA Pol II Ser2P and ICP8_ _HSV-1 (early and late stage)_ < 0.001 (***), *p* value_ICP8 RNA Pol II Ser5P and ICP8 HSV-1 (early and late stage)_ < 0.001 (***)). The data were evaluated with one-way ANOVA method

When viral replication switches from early to late stage, the average distances between ICP8 and RNA Pol II Ser5P change from 195.7 nm to 247.0 nm, with a *p* value < 0.001 (Fig. 7). This distance is smaller than the distance between ICP8 and RNA Pol II Ser2P (Fig. 7, *p* value < 0.05), suggesting ICP8 is positioned closer to RNA Pol II Ser5P than Ser2P. When comparing these values with average distance between ICP8 and viral genome, we found that the distance between ICP8 and HSV-1 genome is always closer than that of ICP8 and RNA Pol II. These differences suggest that viral replication and transcription are partitioned into distinct sub-structures within the replication compartments.

## Discussion

In this study, HSV-1 replication was visualized using super-resolution microscopy. Compared with confocal microscopy, STED showed much better colocalization of two differentially labeled DNA probes directed against the same region of the viral genome. It also detected structural changes from early to late stage of replication, which could not be seen using the confocal method, thus demonstrating that STED is able to discern the fine structures and the dynamic nature of the HSV-1 genome (Figs. 1 and 2). When STED imaging was applied to analyze two probes directed against different, neighboring regions of the viral genome, dynamic changes were observed during the development of viral replication compartments, with viral genomes occupying a smaller space at early stage while a larger space at later stage (Fig. 2e, f, k, l). When the relationship between the viral ICP8 protein and RNA Pol II were examined, we found that ICP8 is closely associated with the viral genome and less associated with RNA Pol II, suggesting that viral replication and transcription are likely portioned into distinct sub-structures within the replication compartments (Figs. 4, 5, 6 and 7). These results demonstrated that STED imaging can reveal details previously unavailable in visualizing replicating HSV-1 genome.

The HSV-1 genome contains two copies of each inverted repeat, TR_L_, IR_L_, TR_S_ and IR_S_, probes located at left hand side of the viral genome (green solid lines, Fig. 2a) is constituted by TR_L_ and IR_L_. As IR_L_ is adjacent to IR_S_, at least part of the signal from probe located at right hand side of the viral genome (red solid line, Fig. 2a) could be interfered by IR_L_ (green) to give a tight associated signal, resulting in higher Rr and *R* values. Thus the data presented represented an underestimation of the spatial expansion of the viral genome during replication. Another parameter *k*, important in colocalization experiments, determines contribution of each antigen in colocalization areas [36]. *k*2, the contribution of DIG, is always higher than *k*1, Biotin’s contribution, which suggested that the efficiency of DIG mixed into newly-synthesized DNA chain may be higher than that of Biotin, or titer of anti-DIG antibody may be higher than that of anti-Biotin antibody. Hence, different mixture efficiencies and various qualities of antibodies might affect signal parameters used to quantify colocalization.

It has been reported that ICP8 regulates viral transcription in two ways: first, by repressing transcription from parental viral genomes [45–47], and sencond by interacting with RNA Pol II and stimulating late gene transcription from progeny DNA templates [38, 39]. ICP8 interacts directly or indirectly with a number of proteins, such as TATA-binding protein-associated factor of 172 kDa (TAF172) and RNA Pol II [38, 48]. However, STED imaging revealed no colocalization between ICP8 and RNA Pol II, and the average distance between ICP8 and RNA Pol II (both Ser2P and Ser5P modified forms) is larger than the distance between viral genome and ICP8, suggesting the primary role of ICP8 is involved in viral genome replication.

We observed a weaker colocalization of the Ser2P modified form of RNA Pol II compared with the Ser5P form in the replication compartments. This is likely due to the fact that HSV-1 viral protein ICP22 rapidly triggers the selective degradation of RNA Pol II Ser2P [49]. On cellular genes, Ser5P levels remain high as RNA Pol II transcribes the first few hundred nucleotides of genes, and as RNA Pol II elongates further downstream, levels of Ser5P drop and Ser2P increase [44]. RNA Pol II Ser2P represents elongating transcription, while RNA Pol II Ser5P stands for new starting transcription. When comparing the relationship between ICP8 and the two modified forms of RNA Pol II, we observed a significant difference, i.e. ICP8 is located further away from the Ser2P than the Ser5P form, suggesting that actively transcribed regions of viral genome (or viral genomes committed to transcription) are placed further away from replicating regions of the viral genome (or replicating viral genomes) than the regions where transcription is new started.

## Conclusions

Here we reported a first observation of replicating HSV-1 genome and its interaction with viral and host proteins at sub-diffraction resolution. We found that the viral genome expands spatially as it enters replication. Viral protein ICP8 tightly interacts with the viral genome, and is organized into sub-structures within the viral replication compartments distinct from host RNA Pol II. These findings suggest that viral replication is a dynamic process and viral genomes, or regions of viral genomes committed to replication and transcription are portioned into different structures within the replication compartments. These findings also suggest that super-resolution microscopy, as represented here by STED, has the potential to unravel much greater details of viral replication process and viral host interactions during lytic HSV-1 infection.

## Methods

### Cells and virus

The human primary fibroblast cells (BJ cells) were obtained from American Type Culture Collection. Cells were grown in Dulbecco’s modified Eagle’s medium (DMEM; Gibco, USA) supplemented with 10 % fetal bovine serum (FBS), penicillin (100 U mL^−1^), and streptomycin (100 μg mL^−1^) in a humidified 5 % CO_2_ atmosphere at 37 °C. 17+ strain of HSV-1 was obtained from Professor Nigel W. Fraser in the Department of Microbiology, Perelman School of Medicine, University of Pennsylvania. The virus was grown and titrated on Vero cells. Viral infections were done according to standard protocols [5]. Briefly, cultured cells were replaced with serum free DMEM, followed by adding the virus and incubating for 1 h with occasional rotation to get an even spread. The culture medium was then replaced by regular DMEM with 10 % FBS and 1 % antibiotics. The HSV-1 cDNA clones and HSV-1 whole genome BAC clone [50] were kindly provided by Professor Chunfu Zheng from the Institute of Biology and Medical Science, Soochow University.

### In situ probes

Components of probes were cut from HSV-1 cDNA clones [51] and mixed equally, labeled with DIG or Biotin in nick translation method. The HSV-1 whole genome BAC clone was labeled with Biotin in nick translation method [52]. Approximately 1 μg DNA was incubated with DNase I and *E. coli* DNA polymerase I at 15 °C for 2 h. A mix of DIG-11-dUTP or Biotin-16-dUTP was added to the reaction to be incorporated into the newly-synthesized DNA chain. Finally the product was incubated at 70 °C for 8 min to deactivate the enzymes.

### Antibodies

RNA Pol II Ser2P polyclonal antibody, RNA Pol II Ser5P polyclonal antibody and ICP8 monoclonal antibody were obtained from Abcam Cambridge (UK). Antibodies against DIG and Biotin were obtained from Roche (Germany) and VECTOR LABORATORIES (USA), respectively. Alexa Fluor® 594 Goat Anti-Mouse IgG (H + L) antibody, Alexa Fluor® 488 Goat Anti-Rabbit IgG (H + L) antibody and Alexa Fluor® 488 Goat Anti-Mouse IgG (H + L) antibody were from Life Technologies (USA).

### FISH

The BJ cells were seeded on glass coverslips in 24-well plates one day before infection and infected at a multiplicity of infection (MOI) of 0.1 PFU/cell or 5 PFU/cell. At 6 h post-infection, cells were fixed with 4 % paraformaldehyde at room temperature for 30 min, extracted with 0.5 % Triton X-100 in PBS for 10 min, deproteinized with 0.1 mol L^−1^ HCl for 10 min and digested RNA with 20 μg mL^−1^ RNaseA for 20 min; Then cells were incubated with probes in hybridization buffer at 95 °C for 4 min; Finally, cells were incubated with antibodies at room temperature for 1 h. Images were acquired using an Olympus FV1000 system (Japan) and Leica TCS SP8 STED 3× (Germany). The distance measuring software was Leica LAS X. Figures were analyzed with Image-Pro Plus 6.0 software (USA).

### IF-FISH

The BJ cells were seeded on glass coverslips in 24-well plates one day before infection and infected at a MOI of 5 PFU/cell. At 6 h post-infection, cells were fixed with 4 % paraformaldehyde at room temperature for 30 min, extracted with 0.5 % Triton X-100 in PBS for 10 min, blocked with 5 % BSA in PBS for 1 h and incubated with primary antibody and secondary antibody for 1 h, respectively. Then cells were deproteinized with 0.1 mol L^−1^ HCl for 7 min, digested RNA with 20 μg mL^−1^ RNaseA for 20 min and incubated with probes in hybridization buffer at 95 °C for 4 min; finally, cells were incubated with antibody at room temperature for 1 h. Images were acquired using a Leica TCS SP8 STED 3× (Germany). The distance measuring software was Leica LAS X. Figures were analyzed with Image-Pro Plus 6.0 software (USA).

### IF

The BJ cells were seeded on glass coverslips in 24-well plates one day before infection and infected at a MOI of 0.1 or 5 PFU/cell. At 6 h post-infection, cells were fixed with 4 % paraformaldehyde at room temperature for 30 min, extracted with 0.5 % Triton X-100 in PBS for 10 min and blocked with 5 % BSA in PBS for 1 h; Then cells were incubated with primary antibodies for 1 h and secondary antibodies for 1 h. Images were acquired using a Leica TCS SP8 STED 3× (Germany). The distance measuring software was Leica LAS X. Figures were analyzed with Image-Pro Plus 6.0 software (USA).

### Statistical analysis

The data were evaluated with the Student’s t-test and one-way ANOVA method, *p* < 0.05 and *p* < 0.001 were considered statistically significant and extremely significant, respectively.

## Abbreviations

BJ cells: human primary fibroblast cells
CTD: carboxyl-terminal domain
DMEM: Dulbecco’s modified Eagle’s medium
FBS: fetal bovine serum
FISH: fluorescence in situ hybridization
HSV-1: herpes simplex virus type 1
ICP8: infected cell protein 8
IF: immunofluorescence
IF-FISH: immunofluorescence-fluorescence in situ hybridization
*k*: antigen contribution
m2: colocalization coefficient
MNase: micrococcal nuclease
MOI: multiplicity of infection
R: overlapping coefficient
RNA Pol II: RNA polymerase II
RNA Pol II Ser2P: serine 2 phosphorylated form of RNA Pol II
RNA Pol II Ser5P: serine 5 phosphorylated form of RNA Pol II
Rr: correlation coefficient
STED: stimulated emission depletion
